# Satiating Capacity and Post-Prandial Relationships between Appetite Parameters and Gut-Peptide Concentrations with Solid and Liquefied Carbohydrate

**DOI:** 10.1371/journal.pone.0042110

**Published:** 2012-07-26

**Authors:** Mieke J. I. Martens, Sofie G. T. Lemmens, Jurriaan M. Born, Margriet S. Westerterp-Plantenga

**Affiliations:** 1 NUTRIM School for Nutrition, Toxicology and Metabolism, Maastricht University Medical Centre + (MUMC+), Department of Human Biology, Maastricht, The Netherlands; 2 Top Institute Food and Nutrition, Wageningen, The Netherlands; Paris Institute of Technology for Life, Food and Environmental Sciences, France

## Abstract

**Background:**

Differences in satiating capacity of liquid and solid meals are unclear.

**Objective:**

Investigating appetite parameters, physiological measurements and within-subject relationships after consumption of a single macronutrient, subject-specific carbohydrate meal in liquefied versus solid form, controlled for energy density, weight and volume.

**Design:**

In a cross-over design, ten male subjects (age = 21.1±3.9 y, BMI = 22.4±1.2 kg/m^2^) consumed a solid (CS, whole peaches +750 ml water) and liquefied carbohydrate (CL, peach blended in 500 ml water +250 ml water) lunch. Appetite profiles, insulin-, glucose- and ghrelin concentrations were measured over three hours. Post-prandial relationships between appetite and blood parameters were calculated using subject-specific regression analyses.

**Results:**

Fullness ratings were higher in the CL (85±5 mm) compared to the CS condition (73±8 mm) at 20 min (p<0.03). Glucose concentrations peaked 20 to 30 min after the start of the lunch in the CL condition, and 30 to 40 min after start of the CS condition. Correspondingly, insulin concentrations were peaked at 20–30 min in the CL condition, and at 30–40 min in the CS condition. AUC or condition x time interactions were not different comparing the CL and the CS condition. Insulin was significantly higher in the CS compared to the CL condition 40 min after the start of the lunch (p<0.05). Fullness scores were significantly related to insulin concentrations but not to glucose concentrations; desire to eat scores were significantly associated with ghrelin concentrations in both, the CL and the CS condition. The relationship between fullness scores and glucose concentrations was not statistically significant.

**Conclusion:**

Liquefied and solid carbohydrate meals do not differ in satiating capacity, supported by appetite profile and relevant blood parameters. Postprandially, fullness and desire to eat were associated with respectively insulin and ghrelin concentrations.

## Introduction

Obesity results from an imbalance of energy intake and energy expenditure [1]. One explanation for the rise in energy intake during the last decade may be the increased consumption of calorically sweetened beverages or other energy-yielding liquids [2], [3]. There has been an undeniable temporal association between the growing consumption of sweetened beverages and the rise in obesity rates, particularly among adolescents and young adults [4]. Physiological mechanisms by which the body senses ingested energy are reported to be less precise for energy contained in liquid beverages as opposed to solid foods [5], [6]. However, the mechanisms underlying the differences in satiation and satiety responses comparing liquid and solid foods have not been clarified yet. There is some evidence that liquids are more satiating than solids [7], [8], [9], [10]. For example, Mattes et al. observed that spoon-ingested soup loads (liquefied apple) led to reductions in hunger ratings comparable to those observed in response to the matching solid food (apples) [11]. Highest hunger rating were observed when the liquid food was drunk (apple beverage) [11]. Those results support the notion that soups have a higher satiety value than beverages, despite their fluid form.

Opposing those results are studies that demonstrate that solid foods are more satiating than liquids [6], [10], [11], [12], [13]. It is suggested that the higher satiety evoked by solid foods is –at least in part- due to the longer oral exposure time caused by chewing [12], [14], while another study suggests that the eating mode of consumption plays a role in the higher satiating value of solids [15]. Still other studies did not find differences in the satiation capacity of beverages and solid foods [16].

The comparison of these studies is often difficult due to the use of different or not comparable foods, i.e. foods that differ along other dimensions than texture evoke responses that cannot solely be ascribed to the food form.

Very few studies so far have included physiological parameters to examine the difference in satiety and satiating capacity of liquid and solid foods. Appetite related biomarkers involved in those differences may include ghrelin-, glucose- and insulin. Haber et al. found that with the rate of ingestion equalized, apple juice was significantly less satiating when compared to apple puree, while apple puree was less satiating than whole apples; both puree and juice condition were associated with disturbances in glucose homeostasis [12]. Zijlstra et al. found that products with similar palatability, macronutrient composition and energy density but different texture lead to significant differences in intake, with the liquid product being consumed more [14]. In contrast to Haber et al., they found no differences in plasma-glucose concentrations [17].

As Lemmens et al. [18] already summarized, possible intra-individual relations between VAS and physiological measurements remain unclear. Some studies show no relation between appetite ratings and ghrelin, glucose or insulin concentrations [19], [20], while others do find associations between appetite ratings and ghrelin or insulin concentration [21], [22]. Studies assessing the relationship between appetite and gut-peptides would greatly benefit from the inclusion of the factor time in a within subject approach [19], [20], [21], [22], [23], [24], [25], [26]. While energy requirements differ between subjects due to differences in body size, muscle mass, gender, etc., changes in appetite parameters do not necessarily have to; a subject feels satiated to a certain extent when his/her subject specific energy requirement is met to a certain extent. This illustrates, why possible relationships between appetite ratings and changes in glucose- and gut-hormone concentrations should be assessed intra-individually when investigating the relationship of appetite ratings and gut peptides.

Taken together, due to the use of a mixture of macronutrients or hardly comparable foods it has been difficult to discriminate between satiating effects of liquid and solid food-textures in the past. Furthermore, the lack of an intra-individual approach to assess the relationship between appetite ratings and physiological measurements made it difficult to explain differences in texture induced satiety.

Therefore, the objective of our study was to investigate differences in appetite profile and in relevant physiological parameters and to study the within-subject relationships between both, comparing a liquefied and solid carbohydrate meal.

## Methods

### Ethics Statement

This study was conducted according to the guidelines laid down in the Declaration of Helsinki and the Medical Ethical Committee of Maastricht University approved all procedures involving human subjects. All subjects were informed on the purpose, procedures and potential risks of the study. Written informed consent was obtained from all subjects.

### Participants

Participants were recruited by advertisements in local newspapers and on notice boards at Maastricht University, the Netherlands. Eleven subjects underwent an initial screening including body weight and height measurements and completed a questionnaire related to medical history, smoking behavior, alcohol consumption, eating behavior and physical activity. Ten healthy male subjects qualified for participation in this study. Power calculation was based on results of related studies. Assuming a mean difference of 14 for satiety ratings, as well as a standard deviation of 14 calculations showed that with an α of 0.05 (power = 1-β = 0.80), at least 10 subjects were needed. Glucose and insulin concentrations were utilized for power calculation in addition to satiety ratings [12]. All subjects were identified as normal breakfast and lunch consumers. Subject characteristics can be found in **Table 1**.

**Table 1:** Participant characteristics.

Characteristics	Mean ± SEM
Male (n)	10
Age (years)	21.1±1.3
Height (cm)	178.5±1.2
Weight (kg)	71.5±1.7
Waist circumference (cm)	77.9±1.1
Hip circumference (cm)	86.2±2.1
BMI^1^	22.4±0.4
BMR^2^	7531.6±29.5

1 Body Mass Index = weight (kg)/height (m)^2^.

2 Basal Metabolic Rate (kJ/day) calculated according to the equation of Harris-Benedict [28].

### Experimental design

**Figure 1:**
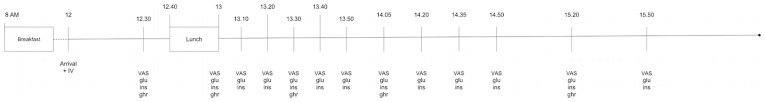
Timeline representing the study design. VAS = visual analog scales on appetite, IV = Intravenous catheter placement, glu = glucose blood sample, ins = insulin blood sample, ghr = active ghrelin blood sample.

The experimental design of the study is depicted in **Figure 1**. The study was conducted in a within-subject, randomized, crossover design. Subjects came to the university twice. Visits were separated by one week. On the day before the trial and during the trial, they were not allowed to consume alcohol or to engage in heavy exercise. On the morning of the test day subjects consumed a predetermined amount of a fluid breakfast at home. After this, subjects were not allowed to eat or drink anything (except water) until arrival at the university at 12 PM. Upon arrival an intra-venous catheter (IV) was inserted for blood sampling. At 12.30, a baseline blood sample for the measurement of plasma insulin, glucose and active ghrelin concentrations was drawn. At 12.40 subjects received a subject-specific (based on individual energy requirements) lunch composed of carbohydrates in a solid or liquefied texture, which they had to consume within 20 min. After lunch, twelve blood samples were drawn for the measurement of plasma insulin, glucose and active ghrelin concentrations. At 4 PM, the catheter was removed and subjects were free to leave the laboratory.

### Meals

#### Breakfast

Subjects had to consume a prescribed breakfast at home, composed of a commercially available breakfast drink called ‘Goedemorgen’ (Campina, The Netherlands). The amount prescribed was based on 20% of the subjects' daily energy requirement (DER), which corresponded to a mean intake ± SEM of 2636.1±43.2 kJ and 840.1±13.8 ml. Daily energy requirements were calculated individually for each of the recruited subjects by multiplying basal metabolic rate (BMR) with the appropriate physical activity factor (1.5–1.8), derived from the Baecke screening questionnaire [27]. The BMR (kJ/day) was calculated according to the equation of Harris-Benedict [28] (**Table 1**).

#### Lunch

Lunch consisted of one of the following two conditions presented to the participant in random order; solid carbohydrate (CS): whole, peeled peaches or liquefied carbohydrate (CL): blended peeled peaches. Peaches consisted of 54% sucrose, 31% fructose, and 15% glucose [29]. The energy content of these meals was subject-specific and controlled for energy density, weight and volume. The solid meal consisted of food and 750 ml water to drink; the liquefied meal consisted of food mixed with 500 ml water and 250 ml water to drink.

The amount of lunch was based on 15% of DER, using the equation of Harris-Benedict and the appropriate physical activity factor [27], [28]. This resulted in a lunch with a mean ± SEM energy content of 1977.6±32.4 kJ and a mean ± SEM weight of 727.0±11.9 grams. The solid meal had to be eaten with knife and fork and the liquefied meal had to be eaten with a spoon in order to equalize rate of ingestion and oral exposure time. All subjects had to finish their meal within 20 min.

The meals were rated for palatability before consumption using visual analogue scales (VAS) by an independent panel of 12 subjects. This panel, was asked how palatable the foods were, after thoroughly experiencing their taste and texture. Palatability was rated on a 100 mm VAS, anchored with ‘not palatable at all’ and ‘extremely palatable’.

### Appetite profile

One hundred mm VAS were used to assess the appetite profile at baseline, and before every blood sample [30]. The scales were anchored with opposing extremes of feelings of fullness, desire to eat and thirst. Subjects were instructed to make a single vertical mark at the appropriate point between the two anchors on each scale to indicate their subjective feelings. These VAS were completed at thirteen time points (at 10 min before the start of the lunch and at 20 min, 30 min, 40 min, 50 min, 60 min, 70 min, 85 min, 100 min, 115 min, 130 min, 160 min, and 190 min after the start of the lunch). All VAS were completed before blood sampling in order to prevent mutual effects.

### Blood sampling

At the beginning of the test session, 40 min before lunch was served (12 PM), a polytetrafluoroethylene catheter was placed in the antecubital vein for blood sampling (BD Venflon™). During each test day, a baseline blood sample was drawn 10 min before the start of the lunch (at −10 min). Subsequently, twelve more blood samples were drawn after the start of the lunch (at 20 min, 30 min, 40 min, 50 min, 60 min, 70 min, 85 min, 100 min, 115 min, 130 min, 160 min, and 190 min) for the measurement of plasma insulin, and glucose concentrations. A blood sample for active ghrelin was drawn (3 ml) five times (at −10 min, 20 min, 50 min, 85 min, 160 min). Blood samples were collected in tubes containing EDTA (BD vacutainer with EDTA, 10 ml) to prevent clotting. Blood plasma was obtained by centrifugation (4°C, 3000 r.p.m., 20 min). All samples were frozen and stored at −80°C until further analysis. Plasma glucose concentrations were analyzed enzymatically by using the hexokinase method (ABX Diagnostics, Montpellier, France); the coefficient of variation (CV) was 0,63%. Plasma insulin concentrations were determined in our own laboratory by means of RIA according to the manufacturer's instructions (Human insulin-specific RIA kit, Millipore, Billerica, Massachusetts, USA); the CV was 3,68%. Plasma concentrations of active ghrelin were measured in our own laboratory by means of radioimmuno assay (RIA) (Human ghrelin (active)-specific RIA kit, Millipore, Billerica, Massachusetts, USA); the CV was 3,70%. Plasma active ghrelin concentrations were measured in acidified plasma with 50 µl of 1 N HCL and addition of 10 µl of phenylmethylsulfonyl fluoride per 1 ml of plasma.

### Statistics

Data were analyzed using StatView 5.0 (SAS Institute Inc., Cary, NC, USA). Areas under the curve were calculated using the trapezoidal method. Differences over time and between conditions (liquefied and solid) were determined using two-factor analysis of variance (ANOVA) with repeated measures. A student's T-test was used to determine the differences between textures at all time points and for the AUC. Following the method previously described by Lemmens et al. [18] the magnitude of the within-subject relationship between changes in VAS scores and blood values were assessed. This method entails the calculation of regression slopes and the regression between VAS scores and blood values for each subject separately, for the corresponding measuring moments (fullness vs. glucose concentrations, fullness vs. insulin concentrations and desire to eat vs. active ghrelin concentrations). After that it is tested if the means of the regression slopes are significantly different from zero (Student's one-sample t-tests) and thus whether the relationships between VAS scores and blood parameter concentrations are statistically significant. Paired Student's t-tests were used to test whether the observed slopes and R^2^ values differed between meal conditions. All tests were two-sided and differences were considered significant at p<0.05. Values are expressed as mean ± standard error of the mean (SEM).

## Results

Measured oral exposure time in the solid and the liquefied condition was not statistically different (19.0±0.3 min and 18.3±0.3 min respectively, P = 0.14).

No statistically significant differences between the palatability ratings of the CS meal (62±4 mm VAS) and the CL meal (62±3 mm VAS) were observed based on the panel ratings preceding the experiment.

### Appetite ratings

**Figure 2:**
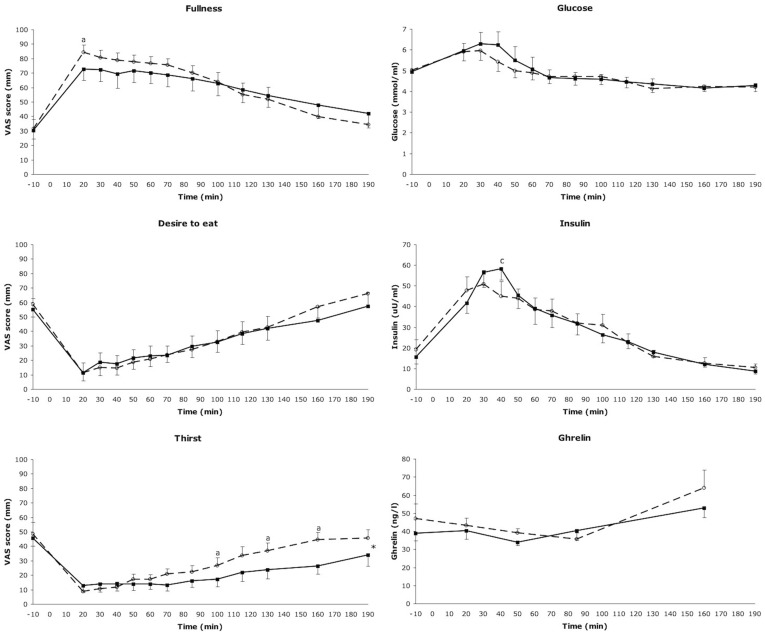
Appetite profiles and blood values. On the left: mean (±SEM) visual analog scale scores for the appetite profiles; hunger, fullness, hunger suppression, desire to eat and thirst for the solid (—▪—) and the liquefied (-○-) condition. On the right: mean (±SEM) plasma glucose, insulin and active ghrelin concentrations for the solid (—▪—) and the liquefied (-○-) condition. **^a^** Indicates a significant difference between the solid and the liquefied condition at that time point (p<0.05). * Indicates significant condition x time interactions (p<0.03).

After lunch fullness ratings significantly increased and desire to eat and thirst ratings significantly decreased over time (p<0.0001). Peak or nadir of the ratings occurred at the end of the lunch, 20 min after the start (**Figure 2**).

There were no statistically significant condition x time interactions for any of the appetite ratings, except for thirst (p<0.03). Moreover, the AUC for the appetite ratings were not different between conditions, except for the thirst ratings where the AUC for thirst was higher in the liquefied condition compared to the solid condition (p<0.05). Fullness was rated higher in the CL condition (85±5 mm) compared to the CS condition (73±8 mm), 20 min after the start of the lunch (p<0.03), when subjects finished eating. Thirst ratings were higher in the CL condition than in the CS condition at 100, 130 and 160 min after the start of the lunch (p<0.03).

### Blood parameters

Blood parameters are represented in **Figure 2**. Time was a significant factor for all blood parameters (p<0.0001). After consumption of the lunch, glucose concentrations peaked 20–30 min after the start of the lunch in the CL condition (5.9±0.3–6.3±0.6 mmol/ml). In the CS condition glucose concentrations peaked at 30–40 min after the start of the lunch (6.3±0.6–6.2±0.6 mmol/ml). Insulin concentrations peaked 20–30 min after the start of the lunch in the CL condition (47.8±6.7–50.9±6.6 uU/ml) and 30–40 min after the start of the lunch in the CS condition (56.6±7.3–58.1±5.4 uU/ml). AUC's of glucose- and of insulin concentrations were not significantly different between conditions and there was no statistically significant condition x time interactions. Insulin concentrations were significantly higher in the CS condition compared to the CL condition at 40 min after the start of the lunch (p<0.05). Active ghrelin concentrations were similar for both conditions; there was no statistically significant condition x time interaction and the AUC was not significantly different between the conditions.

### Relationships between appetite ratings and blood parameters

**Figure 3:**
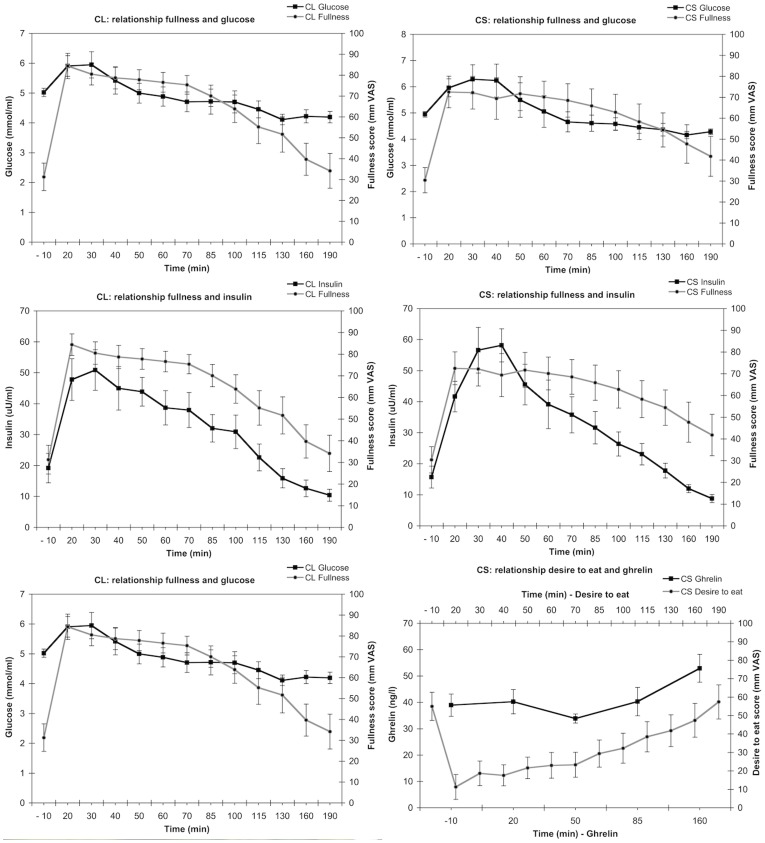
Visual representation of the relationships between the blood values (—▪—) and the visual analog scales (VAS, —•—) in the liquefied (CL) and the solid (CS) conditions.

Following the method by Lemmens et al. [18] physiological intra-individual significant relationships between VAS scores and blood values were assessed, as described in the statistics section (**Figure 3**). Relationships were assessed for fullness vs. insulin concentrations, for desire to eat vs. active ghrelin concentrations and for fullness vs. glucose concentrations. There were no statistically significant differences between the slopes or the R^2^ values comparing meal conditions. The relationship between fullness scores and insulin concentrations was significant in both the CL (slope = 0.8±0.1, p<0.0005, R^2^ = 0.4±0.1) and the CS condition (slope = 0.6±0.2, p<0.005, R^2^ = 0.4±0.1). Similarly, the relationship between ‘desire to eat’ scores and ghrelin concentrations was significant in the CL (slope = 0.9±0.4, p<0.05, R^2^ = 0.3±0.1) as well as in de CS condition (slope = 0.7±0.3, p<0.05, R^2^ = 0.3±0.1). Fullness scores were not related to glucose concentrations in neither condition (CL: slope = 4.2±3.2, p = 0.2; CS: slope = 0.6±4.2, p = 0.9).

## Discussion

The objective of this study was to investigate the relationship of appetite and physiological parameters comparing a carbohydrate meal in liquefied versus solid form, while the meal was controlled for energy density, weight and volume.

Specific attention was given to the within-subject relationships of appetite parameters and glucose-, insulin- and active ghrelin-concentrations. The current study addresses controversies in the existing literature by:

1. giving the same foods in solid and liquefied form with the same amount of total water (750 ml to drink; or 500 ml homogenized +250 ml to drink);
2. utilizing a subject specific design in which subjects received an amount of food bases in their daily energy requirements.

Due to the study design, VAS were controlled for energy requirement and did reflect differences between conditions rather than between subjects. Moreover, when comparing the change in VAS appetite scores with changes in blood parameter concentrations, a statistical approach was used to analyze the possible within subject relationships of these dynamics, including the factor time.

The present study did not find any significant differences in peak-values, AUC or condition x time interactions for fullness and desire to eat. Although insulin reached a significantly lower value in the CL condition at 40 min after the start of the lunch, no significant differences were found for any of the blood parameters comparing AUC's and no condition x time interactions were found. Based on those results, the liquefied carbohydrate meal and the solid carbohydrate meal can be considered as equally satiating, supported by a lack of differences in appetite and relevant blood parameters.

However, despite that overall conclusion, 20 minutes after the start of the lunch, fullness ratings were higher in the liquefied condition compared to the solid condition. A possible explanation for this effect may be that homogenization can influence gastric emptying and thereby increase satiation and satiety ratings more than the fluid and solid components served separately [9], [31]. For example, Santangelo et al. evaluated the effects of the same meal in solid-liquid and in homogenized form on satiety and gastric emptying rate [31]. They found that a vegetable-rich meal was significantly more satiating in a homogenized form than in a solid-liquid state, probably due to a change in gastric emptying time when the food was consumed in the homogenized form [9], [10], [31]. In the current study, a separation of liquid and fluid components for gastric transit may have contributed the delayed peak in glucose and insulin concentrations and the higher insulin concentration in the solid condition compared to the liquefied condition, 40 minutes after the start of the lunch.

The within-subject analysis showed corresponding changes in VAS and insulin and ghrelin concentrations. Desire to eat scores and active ghrelin concentrations declined at the same time, and showed a significant relationship in both meal conditions.

Fullness scores and insulin concentrations increased in parallel and showed a significant relationship in both meal conditions, implying a role for insulin in fullness perception. In a meta-analysis, Flint et al. already showed that the postprandial insulin response is associated with a decrease in hunger and increases in satiety ratings [22]. In contrast to our study results, Zijlstra et al. found that a semi-solid product appeared to be more satiating than a liquid product. However, that finding was not substantiated by a difference in glucose, insulin or ghrelin concentrations [17]. Similar to our results, Haber et al., found similar increases in plasma-glucose concentrations independent of condition and comparable increases in insulin concentrations after fast puree and apples [12].

Studies comparing solid or semi-solid foods often explain their differences in appetite scores by; 1) the need to chew fiber [12] or 2) a longer oral exposure time [32]. The difference with our study is thus the lack of fiber in peeled peaches and the lack of significant differences in oral processing time. The results of our appetite scores in response to the conditions are similar to those of Mattes et al. who found a similar reduction in hunger and increase in fullness in a liquefied meal [11].

Thirst was higher in the liquefied condition as shown by AUC and condition x time interaction. Previous research already showed that drinking water separately with the meal vs. water consumption in the food suppressed thirst more [15]. When thirst is not quenched by water in the meal, this could be due to a different mouth feel, which leads to secondary thirst as described according to Fitzsimons [33], [34].

Although our analyses of regression slopes and R^2^ values showed a relationship between VAS appetite scores and insulin and ghrelin concentrations, the mean explained variation was low (35%). Therefore, we conclude that this relationship is not applicable as a biomarker to the individual level. However, the magnitude of this correlation is sufficient to discriminate intervention-related satiating effects at a group-level.

From a methodological point of view it would have been interesting to additionally use a peach juice as a liquid meal, similar to the use of apple juice in the paper of Mattes et al. [11]. As described in the introduction, they observed the weakest satiety effect in the liquid condition (apple beverage). However, in a drinking condition not only the texture is different but also the mode of consumption, therefore it is difficult to disentangle the cause of observed differences in satiation capacity [15]. The lack of differences in the appetite profile between the liquefied and solid condition in the current study could also be macronutrient specific. Previous studies reported that solid protein evokes a stronger suppression of hunger and desire to eat compared to liquefied or liquid protein [35], [36].

Based on the current results it can be concluded that liquefied and solid carbohydrate meals do not differ in their satiating effects, as evidenced by the lack of differences in appetite profile and relevant blood parameters. Postprandially, fullness and desire to eat were associated with respectively insulin and ghrelin concentrations.
